# Increasing Neonatal Mortality among Palestine Refugees in the Gaza Strip

**DOI:** 10.1371/journal.pone.0135092

**Published:** 2015-08-04

**Authors:** Maartje M. van den Berg, Haifa H. Madi, Ali Khader, Majed Hababeh, Wafa’a Zeidan, Hannah Wesley, Mariam Abd El-Kader, Mohamed Maqadma, Akihiro Seita

**Affiliations:** 1 Health Department, Headquarters, United Nations Relief and Works Agency for Palestine Refugees in the Near East (UNRWA), Amman, Jordan; 2 Regional Office for the Eastern Mediterranean, World Health Organization, Cairo, Egypt; 3 Health Department, Field Office Gaza, UNRWA, Gaza City, Palestinian Territory; Queensland University of Technology, AUSTRALIA

## Abstract

**Background:**

The United Nations Relief and Works Agency for Palestine refugees in the Near East (UNRWA) has periodically estimated infant mortality rates among Palestine refugees in Gaza. These surveys have recorded a decline from 127 per 1000 live births in 1960 to 20.2 in 2008.

**Methods:**

We used the same preceding-birth technique as in previous surveys. All multiparous mothers who came to the 22 UNRWA health centres to register their last-born child for immunization were asked if their preceding child was alive or dead. We based our target sample size on the infant mortality rate in 2008 and included 3128 mothers from August until October 2013. We used multiple logistic regression analyses to identify predictors of infant mortality.

**Findings:**

Infant mortality in 2013 was 22.4 per 1000 live births compared with 20.2 in 2008 (p = 0.61), and this change reflected a statistically significant increase in neonatal mortality (from 12.0 to 20.3 per 1000 live births, p = 0.01). The main causes of the 65 infant deaths were preterm birth (n = 25, 39%), congenital anomalies (n = 19, 29%), and infections (n = 12, 19%). Risk factors for infant death were preterm birth (OR 9.88, 3.98–24.85), consanguinity (2.41, 1.35–4.30) and high-risk pregnancies (3.09, 1.46–6.53).

**Conclusion:**

For the first time in five decades, mortality rates have increased among Palestine refugee newborns in Gaza. The possible causes of this trend may include inadequate neonatal care. We will estimate infant and neonatal mortality rates again in 2015 to see if this trend continues and, if so, to assess how it can be reversed.

## Introduction

Infant mortality rates and child mortality rates are widely regarded as sensitive (proxy) measures of the health status not only of children, but of whole populations.[1] The United Nations Millennium Development Goal 4 (MDG4) target of reducing under-five child mortality by two thirds from 1990 to 2015 has captured substantial political, donor, and country focus over recent decades.[2,3] This global goal is mainly motivated by huge disparities in child mortality between and within nations, but also by the evidence that, despite the inverse relationship between infant mortality and socioeconomic status,[4,5] mortality can be reduced even in low-income countries.[6] Indeed, child mortality has been decreasing in many countries for many decades.[3] However, to judge by infant and child mortality rates, important inequality exists across countries.

One example is the disparity in these measures between Israel and the occupied Palestinian territory. In 2013, the infant mortality rate was estimated at 3.5 per 1000 live births in Israel, compared with 15.8 per 1000 live births in the occupied Palestinian territory.[3] In particular, the socioeconomic condition in the Gaza Strip (hereafter noted as ‘Gaza’) has deteriorated dramatically following imposition of a blockade by the Israeli government in 2007.[7] The blockade has impacted the health sector in Gaza, as hospitals continue to lack adequate physical infrastructure, drugs and supplies.[8,9] In addition, armed conflicts during June 2006, December 2008 to January 2009, November 2012 and July and August 2014 have contributed to a health and healthcare environment that may have affected pregnancy outcomes and the health care provided to infants.

The United Nations Relief and Works Agency for Palestine refugees in the Near East (UNRWA) delivers primary maternal and child health care to Palestine refugees and has periodically estimated infant mortality rates among Palestine refugees in Gaza. These surveys have recorded a decline from 127 per 1000 in 1960, to 82 per thousand in 1967, to 33 per thousand in 1995, to 20.2 per thousand in 2008.[8,10,11] Regular estimation of infant mortality among UNRWA’s beneficiaries is important for monitoring the Agency’s progress towards achieving MDG4. As a follow-up to previous UNRWA estimates we have extended the series by conducting a survey in 2013 to estimate infant mortality in 2011.

## Methodology and Methods

### Ethical considerations

Informed consent from participants was obtained by verbal consent. A reasonable number of Palestine refugees is illiterate or partially literate therefore verbal consent was considered more suitable than written consent. After verbal explanation of the purpose of the research and the content of the interview, participant’s consent was documented on the data collection sheet. The Ethics Office of United Nations Relief and Works Agency at Headquarters Amman in Jordan approved the research proposal and the consent procedure.

### Preceding-birth technique

This article, presenting data from Gaza, is part of a larger study conducted within four of UNRWA's five fields of operation (Gaza, West Bank, Jordan, and Lebanon). To ensure comparability with previous data, the preceding-birth technique was used.[10–12] Multiparous mothers who attended UNRWA health centres with their most recently-born child for registration and immunization were interviewed and asked if their preceding child was alive or dead. The preceding-birth technique is derived from the original Brass-Macrae method [13] and was modified by asking the age at death, categorized as early-neonatal (≤7 days), late-neonatal (8-≤28 days), and post-neonatal (>28 days-1 year).[11,14] This technique is useful to estimate infant mortality rates indirectly in a population with generally high-fertility rates and short birth intervals.[12,15] It is valuable to measure changes in mortality rates as one element in the regular evaluation of UNRWA practices.[12]

Currently, no appropriate registration exists for infant deaths among Palestine refugees in Gaza. The preceding-birth technique is an alternative to prospective analysis.[15] Household surveys are unfeasible as many Palestine refugees live outside refugee camps and are therefore difficult to target.[16] The preceding-birth technique can be used at (medical) service points widely used by the population. For more than two decades over 95% of registered Palestine refugee infants receive child health care, including immunization, at UNRWA health centres,[16] so our sample can be considered representative of the population served by UNRWA.

The mortality rate is estimated by dividing the number of dead preceding children by the total number of preceding children (including twins and triplets). Mothers of whom the most recently-born child died would not attend the clinic and so will not be included in the survey. Similar to previous surveys and as described by Hill and Aguirre, the estimated mortality rates are adjusted by a correction factor of 1.09, to account for mothers who lost their current child in addition to the mothers who lost both the current and the preceding child.[11,12]

### Study population

The target population included multiparous mothers with at least two children born alive, who attended one of the 22 UNRWA health centres in Gaza for registration and immunization of their most recently-born child. The following assumptions and requirements were used to calculate the sample size by the Epi-info StatCalc program for population surveys: based on 2008 data, the estimated infant mortality rate in Gaza was 20.2.[10] A 95% confidence interval of ± 5 deaths per 1000 live births was desired. The estimated sample size needed was 3130 mothers, and was stratified in proportion to the number of registered newborn infants per year at each health centre.

Mothers with only one child and mothers whose most recently-born child was a stillbirth were excluded from this study as the preceding-birth technique concerns the survival or mortality of children born alive. If the preceding pregnancy resulted in miscarriage, information was obtained on the child born before the miscarriage. Enrolment of respondents began on 15 August 2013 and ended on 28 October 2013, when the desired sample size had been reached. The response rate was 99%.

### Data collection

A standardized questionnaire was used to retrieve the required information through face-to-face interviews of mothers, conducted by trained, experienced nurses. The questionnaire included demographic and social variables. Child health records and antenatal records were used to supplement the information provided by the interviewees. A few variables from these records were added to the survey used in 2008, including gestational age and weight at birth of preceding child. The gestational age was calculated by using the first day of the last menstrual period of the mother. In addition, information about the pregnancy leading to the birth of the preceding child was added. Pregnancies were categorized by medical staff at the last antenatal visit before delivery as: normal (no risk factor), alert-risk (one risk factor), or high-risk (2 or more risk factors).

Each preceding child was documented as either currently alive or dead. The senior staff nurse and the doctor in charge jointly collected the information on the direct cause of death. According to the mother’s report and documentation in the child’s health record, the cause of death was categorized as: preterm birth, birth complication, congenital anomaly or metabolic disease, infection, injury or accident, other (including sudden infant death syndrome), and unknown.

### Statistical analysis

SPSS version 22.0 was used for statistical analysis. The method of mortality rate estimation was described in the preceding-birth technique section. Fisher’s test was used to compare the difference in mortality rate between the survey conducted in 2013 and the survey conducted in 2008. The causes of death are presented descriptively.

By using the preceding-birth technique, we calculated the birth-interval between preceding child and the most recently-born child, but not the birth-interval before the preceding child. This interval was used to determine the period of time the mortality data refer to. The reference time of the survey depends on this birth-interval and the age at registration of the most recently-born.

To assess determinants of infant death, multiple logistic regression was used. Categorization of continuous independent variables was performed based on previously literature: maternal age <18 years, 18–34 years, or 35 years or older; number of pregnancies ≤3, 4–5, or ≥6; maternal education 12 years (high-school) or more than 12 years; birth-interval less than 24 months or 24 months or more [17]; gestational age preterm birth (<37 weeks) or term birth; birth weight low birth weight (<2500 grams) or more than 2500 grams.

All independent variables were tested for collinearity, confounding and interaction. All measured confounding variables were included in the model. Collinearity was found between the number of living children, number of children born and number of pregnancies. Only the number of pregnancies was included in the final model as this included miscarriages and stillbirths and was considered clinically most relevant. Interaction was found between gestational age and birth weight. The same method was used to develop multiple logistic regression models to evaluate risk factors for preterm birth and death due to congenital anomaly.

## Results

### Mortality rates

**Fig 1:**
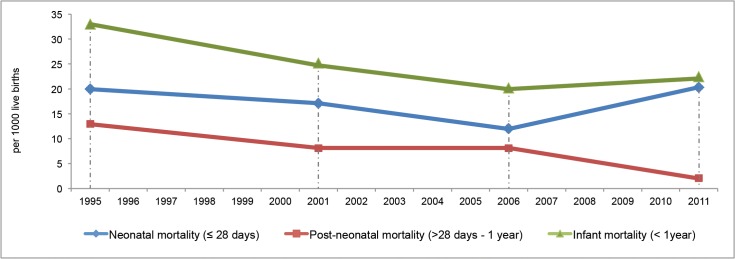
Trend of mortality rates. Infant, neonatal and post-neonatal mortality rate per 1000 live births among Palestine refugees in Gaza (Sources UNRWA surveys^10^ conducted in 1997, 2003, 2008 and 2013, with reference times of 1995, 2001, 2006 and 2011, respectively).

Table 1 shows the demographics of the study population in 2013, which are comparable to those of the population studied in 2008. The mean birth-interval was 32.8 (SD 21.8, range 9–165) months) and the mean age at registration of the most recently-born was 8.7 (SD 6.5, range 1–205) days, so the reference time for the 2013 survey was November 2011. The mortality rates over time are presented in Fig 1. When compared to the 2008 survey, there were changes in infant mortality by 2.2 per 1000 live births (95% CI 0.6–3.8) and statistically significant increase of neonatal mortality by 8.2 per 1000 live births (5.1–11.3). Post-neonatal mortality rate declined statistically significant by 6.1 per 1000 live births (3.4–8.8), see Table 2. Of all 65 infant deaths, the proportion of early-neonatal deaths was 46% (n = 30), late-neonatal deaths 44% (n = 29) and post-neonatal deaths 9.2% (n = 6).

**Table 1:** Study population.

Year of survey		2008	2013
Newborn under registration		n = 3706	n = 3128
Sex–male		1895 (51%) 49.5%-52.7%	1566 (50.1%) 48.3%-51.9%
Age at registration in days		8.1 (±5.2)	8.7 (±6.5)
**Mother**	** **	**n = 3706**	**n = 3128**
Age in years		28.5 (±5.9)	27.8 (±5.5)
Education in years		11.5 (±3.2)	12.6 (±3.1)
Number of pregnancies		5.1 (±2.8)	4.6 (±2.6)
Number of children born alive		4.7 (±2.2)	4.0 (±2.0)
Living inside camp		no data	1038 (33.4%) 31.8%-34.9%
Working mothers		no data	287 (9.2%) 8.2%-10.4%
Consanguineous marriage		no data	960 (30.7%) 29.1%-32.4%
Risk classification preceding pregnancy	normal	no data	2014 (64.4%) 62.7%-66.0%
	alert	no data	657 (21.1%) 19.7%-22.5%
	high	no data	449 (14.4%) 13.2%-15.7%
**Preceding child**	** **	** **	**n = 3162**
Gestational age in weeks		no data	39.3 (±1.7)
Birth weight in grams		no data	3262.9 (±528.0)
Preterm birth (< 37 weeks)		no data	157 (5.0%) 4.2%-5.7%
Low birth weight (< 2500 gram) and preterm		no data	86 (2.7%) 2.2%-3.3%
Low birth weight and full term		no data	90 (2.8%) 2.2%-3.4%

Data are presented as mean (±SD) or n (%) with 95% confidence intervals.

**Table 2:** Infant mortality compared to previous survey.

Study conducted	2008	2013	
Reference time of the survey	January 2006	November 2011	
	Mortality rate per 1000 live births (95% CI)		Change per 1000 live births (95% CI)
**Early neonatal mortality (0–7 days)**	no data	10.3 (6.5–14.2)	.
**Late neonatal mortality (8–28 days)**	no data	10.0 (6.5–14.2)	.
**Neonatal (≤28 days)**	12.1 (8.7–16.4)	20.3 (15.3–26.2)	8.2 (5.1–11.3)^*^
**Post-neonatal mortality (29 days—1 year)**	8.2 (5.5–10.9)	2.1 (1.1–3.3)	-6.1 (-3.4–-8.8)^*^
**Infant mortality (<1 year)**	20.2 (15.3–25.1)	22.4 (16.4–28.3)	2.2 (0.6–3.8)

^*^ p< 0.05 mortality rate of 2013-survey compared to 2008-survey.

### Predictors of infant death

Multiple logistic regression analysis showed that preterm birth (OR 9.88, 95% CI 3.98–24.85) and consanguinity (2.41, 1.35–4.30) were predictors of infant death (Table 3). Mothers with high-risk pregnancies had higher odds of losing their baby (3.09, 1.46–6.53). In addition, a birth interval of less than 24 months was related to infant death (3.98, 2.17–7.30).

**Table 3:** Risk factors of preterm birth and infant death.

		Preterm birth	Infant death
Independent variables		*Adjusted odds ratio (95% CI)*	*Adjusted odds ratio (95% CI)*
**Age of mother at preceding birth**	18–34 years	reference	reference
	< 18 years	1.38 (0.59–3.24)	0.17 (0.02–1.52)
** **	≥ 35 years	0.65 (0.27–1.59)	1.07 (0.33–3.48)
**Maternal education–less than 12 years**		0.73 (0.48–1.11)	1.81 (0.98–3.44)
**Number of pregnancies **	≤ 3	reference	reference
	4–5	0.74 (0.48–1.14)	1.45 (0.70–2.98)
	≥ 6	0.72 (0.45–1.15)	1.32 (0.62–2.84)
**Maternal employment**		0.79 (0.41–1.51)	1.18 (0.40–3.55)
**Residence inside camp **		0.99 (0.69–1.43)	1.04 (0.57–1.89)
**Consanguinity**	** **	1.16 (0.80–1.68)	2.41 (1.35–4.30)
**Risk classification preceding pregnancy**	normal	reference	reference
	alert	1.82 (1.19–2.78)	1.61 (0.79–3.29)
	high	2.94 (1.86–4.64)	3.09 (1.46–6.53)
**Twin/triplet pregnancy**		9.54 (4.46–20.38)	1.32 (0.36–4.83)
**Birth-interval below 24 months**		0.99 (0.69–1.42)	3.98 (2.17–7.30)
**Sex preceding child (female)**		0.86 (0.61–1.22)	1.65 (0.93–2.94)
**Preterm birth (< 37 weeks)**		* *	9.88 (3.98–24.85)
**Low birth weight (< 2500 grams) **			3.20 (0.93–11.04)
**Preterm birth by low birth weight (interaction)**			1.20 (0.24–5.60)

Hosmer and Lemeshow test and classification preterm birth: p = 0.98, 75.0%; infant death p = 0.09, 82.7%.

### Causes of death

The main causes of infant deaths were preterm birth and congenital anomalies (Table 4). No differences in causes of death were identified when compared to the UNRWA survey in 2008.[17]

**Table 4:** Causes of infant death in current and previous survey.

Year of survey	2008	2013
Number of death cases	69	65
Preterm birth	17 (25%)	25 (39%)
Birth complication	2 (2.9%)	2 (3.1%)
Congenital malformation/metabolic disorder	21(30%)	19 (29%)
Infections	14 (20%)	12 (19%)
Accidents/injuries	0	0
Other (including sudden infant death syndrome)	15 (22%)	7 (11%)

Multiple logistic regression analysis showed that mothers classified as having alert-risk pregnancies had higher probability of preterm birth than mothers with normal pregnancies (OR 1.82, 95% CI 1.19–2.78) as did mothers with high-risk pregnancies (OR 2.94, 1.86–4.64). In addition, twin/triplet pregnancy was related to preterm birth (OR 9.54, 4.46–20.38), see Table 3.

Cardiac anomalies and neurological malformations were most common among deaths due to congenital anomalies and metabolic disorders. After adjusting for social factors and maternal age in a separate regression model, consanguineous marriage showed high odds for death due to congenital anomaly or metabolic disorder (OR 3.38, 95% CI 1.44–7.92).

## Discussion

This survey was conducted in 2013 with November 2011 as a reference time. It showed for the first time in five decades that mortality rates had increased among Palestine refugee newborns in Gaza. This finding is worrying and suggests that Gaza is currently not on track to meet the 2015 MDG4 under-five mortality target of 14 per 1000 live births.[18] However, post-neonatal mortality rate declined significantly and the infant mortality rate was only slightly higher. These estimates are based on small numbers of deaths, and the confidence intervals are wide, so the infant mortality rate could in fact be stable or continuing to decline.

The preceding-birth technique was developed in principle to detect relative changes in mortality rates, as an alternative to measuring absolute levels.[12] We chose to use the same methodology as previous UNRWA surveys to achieve consistency in estimating mortality trends. Reassessment of the mortality rates in 2015 is required to extend our findings. In addition, UNRWA will work with others to establish prospective registration of infant births and deaths in Gaza. As UNRWA recently introduced electronic medical records in Gaza, follow-up of all registered births should become easier.

Apart from the relatively small number of deaths upon which our estimates of infant mortality are based, there are some other limitations of our indirect method for estimating mortality rates. The data collected depend on mothers’ recall, which may have been affected by recall bias. Preceding stillbirths were not included in this survey and it is possible that some early-neonatal deaths and stillbirths may have been mixed-up. Further, we acknowledge that mothers’ recall of the cause of death may have been inaccurate or the documentation in the child health record may have been incomplete. Identifying the correct cause of death in newborns and infants is challenging and therefore verbal autopsy is widely used to improve the registration of cause of death.[19] This methodology was not used during UNRWA surveys. Another drawback is that mothers with only one child were not included in this study, which could have resulted in underestimation of infant mortality as primiparous women are more likely to experience perinatal mortality.[20] However, this was the same methodology as in previous surveys and will only be a significant bias if a large proportion of mothers stop childbearing after a single birth; this is not common in Gaza where the fertility rate is around five births per woman.[21] Another limitation is that the population studied in 2013 could be different compared to the population that was studied in 2008. This potential variation was not identified with the available data, as presented in Table 1. However, some variables, such as gestational age and birth weight, were not collected during the 2008 survey.

Our results reveal an increase in deaths within four weeks of live birth. We estimated that these neonatal deaths accounted for 90% of all infant deaths, which is higher than the approximately 60% documented elsewhere.[3,18] It is possible that mother’s memory of the exact age at death was inaccurate and that some deaths were recorded as having occurred within a month after birth although they actually happened later in infancy. However, this would also have been the case for the previous surveys.

Several interventions exist to prevent neonatal deaths, the most effective of which are related to care during labour followed by care provided to the neonate after birth.[22] In Gaza, almost all deliveries take place in hospitals under skilled supervision,[23] but, as was previously reported by the United Nations Population Fund, Medical Aid For Palestinians, and the World Health Organization, the quality of obstetric and neonatal care could be improved.[24–26]

These evaluations showed that there were gaps in health care infrastructure, shortage of (transport) incubators, inadequate practices on infection prevention, insufficient support for breastfeeding and shortages of life saving drugs, such as surfactant. In addition, a need for Neonatal Life Support training and standardized (resuscitation) protocols was noted. These findings, coupled with ours suggest that effective interventions to prevent deaths among preterm born infants may not have been used optimally.[24–26]

UNRWA provides only primary health care so it is relevant to evaluate potentially effective interventions such as family planning, preconception, antenatal and postnatal care. Promoting optimal birth spacing by family planning services can help to reduce infant mortality[27,28] and modern contraceptive methods are increasingly used among Palestine refugee families.[17] However, compared to the previous survey in 2008, the mean birth-interval declined from 36.8 months to 32.8 months. By using the preceding-birth technique, we calculated the birth-interval between preceding child and most recently-born child, and not the birth-interval before the preceding child. Since, birth spacing is an important determinant of reproductive behaviour, we included it as potential confounder in the models to determine potential risk factors for preterm birth and infant death. A birth-interval after the preceding child was born of less than 2 years was associated with infant death. Short birth-interval could therefore be an effect of losing the baby and not necessarily a causal predictive factor. An evaluation of current contraceptive practices among the Palestine refugee population is planned to identify how family planning services can be improved.

Preconception care was integrated in UNRWA’s maternal and child health program in 2010,(16) and includes daily folic acid supplementation before pregnancy and in the first trimester to prevent neural tube defects.[29] Among the pregnant Palestine refugee women, almost all have at least four antenatal visits during pregnancy, with an average of 6.8 visits.[16] Women at risk of complications during pregnancy or delivery, for example due to pregnancy-induced hypertension, or with symptoms of pre-eclampsia, gestational diabetes mellitus or anaemia are identified during these antenatal visits to intensify follow-up, start appropriate treatment, or referral to a specialist. Our data show that women classified with high-risk pregnancy had a higher odds of losing the baby.

Antenatal corticosteroid administration to reduce complications and deaths from being born immature [30] is currently not standard care in UNRWA primary care settings, but it is possible that this effective intervention was not used optimally in secondary care. Other effective interventions, such as prompt management of neonatal asphyxia and appropriate administration of surfactant, may also not have been used optimally.

Postnatal visits occur at UNRWA clinics in Gaza in the first two weeks of life. During these visits screening for congenital anomalies and metabolic disorders, such as phenylketonurie, hypothyroidism and glucose-6-phosphate dehydrogenase deficiency, occurs and counselling is given on exclusive breastfeeding and newborn care. Exclusive breastfeeding may prevent infection-related neonatal deaths [31] and sudden infant death syndrome.[32] In Gaza, only about a third of the infants under 6 months of age are exclusively breastfed,[23] which indicates that efforts should be made to increase this rate, for which community-based interventions may be useful.[33]

In conclusion, we have estimated that, for the first time in five decades, the mortality rate has increased among Palestine refugee newborns in Gaza, and this may reflect inadequate neonatal care in hospitals. Finally, we note that our study related to a period before the most recent and severe of four armed conflicts in Gaza during the past years. We will estimate infant and neonatal mortality rates again in 2015 to see if this trend continues. In the meantime, UNRWA will work with others to assess how to act on our findings, for example, by enhancing public awareness on the negative health consequences of consanguinity, promoting the positive effects achievable with family planning services, and ensuring effective antenatal care for women with high-risk pregnancies.
